# Evaluation of the Bitterness of Traditional Chinese Medicines using an E-Tongue Coupled with a Robust Partial Least Squares Regression Method

**DOI:** 10.3390/s16020151

**Published:** 2016-01-25

**Authors:** Zhaozhou Lin, Qiao Zhang, Ruixin Liu, Xiaojie Gao, Lu Zhang, Bingya Kang, Junhan Shi, Zidan Wu, Xinjing Gui, Xuelin Li

**Affiliations:** 1 Institute of Clinical Pharmacy, Beijing Municipal Health Bureau, Beijing 100035, China; linzhaozhou@gmail.com; 2 School of Chinese Materia Medica, Beijing University of Chinese Medicine, Beijing 100102, China; zhangqiao@bucm.edu.cn; 3 Department of Pharmacy, The First Affiliated Hospital of Henan University of Traditional Chinese Medicine, Zhengzhou 450000, China; s.summer.lulu@live.com (L.Z.); kangby2008@163.com (B.K.); sjhx0@126.com (J.S.); xuelinli450000@163.com (X.L.); 4 The Level Three Laboratory of Chinese Traditional Medical Preparation of State Administration of TCM, Zhengzhou 450000, China; 5 Key Laboratory of Viral Diseases Prevention and Treatment of TCM of Henan Province, Zhengzhou 450000, China; 6 School of pharmacy, Henan University of Traditional Chinese Medicine, Zhengzhou 450008, China; gaoxiaojie1990@126.com (X.G.); zidan_wu@my.uri.edu (Z.W.); guixinjing1991@163.com (X.G.); 7 Department of Computer Science and Statistics, University of Rhode Island, Kingston, RI 02881, USA

**Keywords:** electronic tongue, robust partial least squares, bitterness evaluation, sensors, outlier detection

## Abstract

To accurately, safely, and efficiently evaluate the bitterness of Traditional Chinese Medicines (TCMs), a robust predictor was developed using robust partial least squares (RPLS) regression method based on data obtained from an electronic tongue (e-tongue) system. The data quality was verified by the Grubb’s test. Moreover, potential outliers were detected based on both the standardized residual and score distance calculated for each sample. The performance of RPLS on the dataset before and after outlier detection was compared to other state-of-the-art methods including multivariate linear regression, least squares support vector machine, and the plain partial least squares regression. Both R^2^ and root-mean-squares error (RMSE) of cross-validation (CV) were recorded for each model. With four latent variables, a robust RMSECV value of 0.3916 with bitterness values ranging from 0.63 to 4.78 were obtained for the RPLS model that was constructed based on the dataset including outliers. Meanwhile, the RMSECV, which was calculated using the models constructed by other methods, was larger than that of the RPLS model. After six outliers were excluded, the performance of all benchmark methods markedly improved, but the difference between the RPLS model constructed before and after outlier exclusion was negligible. In conclusion, the bitterness of TCM decoctions can be accurately evaluated with the RPLS model constructed using e-tongue data.

## 1. Introduction

The sensation of taste can be divided into five basic tastes: sweetness, sourness, saltiness, bitterness, and umami [1], with bitterness being the most difficult to tolerate. Unfortunately, most active pharmaceutical ingredients taste bitter [2]. According to the 2010 Chinese Pharmacopoeia, 49.0% of herbs or decoction pieces are bitter. Humans identify various tastes and experience different gustatory reactions by generating a series of electrical signals based on molecules in the mouth, and transmitting them to the corresponding brain area to be distinguished. The primary quantitative method for determining bitterness intensity is the traditional human taste panel method (THTPM) [3,4,5,6,7,8]. However, this technique has multiple challenges, including the use of human volunteers who may be exposed to dangerous specimens or suffer tester fatigue [9]. Therefore, the analytical taste-sensing multichannel sensory system called the electronic tongue (e-tongue), which can be used to safely and affordably assess taste, has replaced sensory panelists.

Advances in the field of multi-sensor and multi-dimensional data analysis offer a powerful means to measure and analyze information on complex systems. The e-tongue is an analytical instrument with an array of nonspecific, low-selective, chemical sensors with high stability and cross-sensitivity to different species in solution. When gustatory substances are absorbed in the film, the data are sensitively obtained by potential changes to the membrane. Taste-quality perception and recognition are based on building or recognition of activated sensory nerve patterns by the brain, which is achieved by the e-tongue’s statistical software that interprets the sensor data into taste patterns. The result is detection of taste similar to the human palate [1,10]. E-tongue technology has been applied to the food industry for decades [11,12], and is primarily used for food traceability [13], food freshness [14], food quality [15,16], and safety testing [17]. Concurrently, this technology has been gradually applied to TCM for discrimination, classification [18,19,20,21], and taste-masked studies [22], as well as the bitterness evaluation of TCMs [23]. However, using the e-tongue and appropriate chemometrics methods as a basis for quantitative study of bitterness in different Traditional Chinese Medicines decoctions, which contain different types of bitter components, has not been reported. Besides, studying and evaluating their bitterness have great pharmaceutical significance, as inactive ingredients that have a major contribution to the bitter taste could be removed, and sweeteners and additives might be added to make it more palatable.

Gustatory evaluation using the e-tongue can only be realized by performing a regression analysis between the tastes collected from the THTPM and sensor data acquired by the e-tongue system. There are a variety of modeling methods used to describe this relationship such as the back propagation neural network (BPNN) [24], partial least squares (PLS) regression [25], and support vector machine (SVM) [26], as well as their modifications such as the Gaussian belief propagation model [27] and least squares support vector model (LSSVM) [28]. However, these methods are sensitive to unavoidable outliers in real applications. Therefore, to achieve a robust evaluation model, the robust partial least squares (RPLS) regression that is more resilient to outliers than other methods [29,30], was adopted in this study to quantify the bitterness of TCMs. A comparison study performed with state-of-art methods clearly demonstrated its robustness and prediction accuracy. In addition, the prediction performance of the benchmark methods was enhanced after potential outliers were removed from the training set.

## 2. Materials and Methods

### 2.1. Apparatus

The E-tongue experiment was performed using an Astree II electronic tongue (Alpha M.O.S, Toulouse, France). The E-tongue consisted of a hexadecimal autosampler, an Ag/AgCl reference electrode, a data acquisition system, an AlphasoftV12 workstation, and seven sensors (ZZ2808-2-512, CA2804-2-440, DA2808-12-330, BA2808-2-230, GA2808-2-361, BB2011-09-141, AB2011-10-010, which were abbreviated to ZZ, CA, DA, BA, GA, BB, and AB, respectively). The seven sensors were specifically developed for bitterness detection. BSA224S-CW electronic balance (Sartorius Scientific Instruments Co., Ltd., Beijing, China), HK250 desktop ultrasonic cleaner (Shanghai Kedao Ultrasonic Instrument Co., Ltd, Shanghai, China), AM-5250B magnetic stirrer (Changzhou Renhe Instrument Factory, Jintan, China), PHS-3C pH meter (Shanghai Second Analytical Instrument Factory, Shanghai, China), and JXJ-II tabletop centrifuge (Shanghai Anting Scientific Instrument Factory, Shanghai, China) were also used. The software used included MATLAB R2011b (Mathworks Inc., Natick, MA, USA), the RSIMPLS toolbox from the MATLAB Library for Robust Analysis (LIBRA) toolbox [31], and the LS-SVMlab Toolbox (version 1.8) [32].

### 2.2. Selection of Concentrations

Bitterness was divided into five levels according to the literature [27], and each level was given a range of values. Berberine hydrochloride solutions at various concentrations were used as references [23]. The concentrations were obtained by pre-testing different concentrations of the berberine hydrochloride reference solution (Table 1).

**Table 1:** Bitterness rank and concentration of corresponding reference samples.

No.	Description of Intensity of Bitterness	Rank Assigned	Corresponding Scale	Conc. of Corresponding Reference Samples
1	Imperceptible	I	[0.5–1.5)	0 mg/mL (0 mM)
2	Slight	II	[1.5–2.5)	0.01 mg/mL (0.027 mM)
3	Moderate	III	[2.5–3.5)	0.05 mg/mL (0.134 mM)
4	High (but still acceptable)	IV	[3.5–4.5)	0.1 mg/mL (0.269 mM)
5	Extreme (almost unacceptable)	V	[4.5–5.5]	0.5 mg/mL (1.344 mM)

### 2.3. Sample Preparation

A total of 35 herbs from the Chinese Pharmacopoeia, variously characterized as tasteless, slightly bitter, bitter, and extremely bitter, were selected for the experiments (Table 2). All of the TCM samples, including Clematidis Armandii Caulis, Xanthii Fructus, Corydalis Rhizoma, and Sophorae Flavescentis Radix (batch number: 20120224), were purchased from Zhongyi Pharmaceutical Co., Ltd. (Zhengzhou, China) For details please refer to Table 2. The berberine chloride monomer was purchased from Yuxin Pharmaceutical Co., Ltd. (Chengdu, China; batch number: 101002). All of the other reagents were of analytical grade and created with purified water.

**Table 2:** The 35 samples and their degree of bitterness based on human testing.

No.	Drug (The Latin Name)	Drug (Chinese Pinyin)	Human Test Data	pH
			Bitterness Intensity	
1	CLEMATIDIS ARMANDII CAULIS	Chuanmutong	0.70 ± 0.24	6.71
2	MORI RAMULUS	Sangzhi	0.67 ± 0.23	6.57
3	UNCARIAE RAMULUS CUM UNCIS	Gouteng	0.70 ± 0.25	6.72
4	PLANTAGINIS SEMEN	Cheqianzi	0.71 ± 0.20	5.82
5	BAMBUSAE CAULIS IN TAENIAS	Zhuru	1.24 ± 0.38	6.80
6	CHANGII RADIX	Mingdangshen	0.73 ± 0.30	6.45
7	LYCOPI HERBA	Zelan	1.19 ± 0.62	6.20
8	PORIA	Fuling	0.63 ± 0.17	6.74
9	TETRAPANACIS MEDULLA	Tongcao	0.64 ± 0.14	7.15
10	XANTHII FRUCTUS	Cang’erzi	1.21±0.50	6.81
11	EUCOMMIAE CORTEX	Duzhong	1.26 ± 0.57	6.15
12	ALISMATIS RHIZOMA	Zexie	0.95 ± 0.50	7.14
13	PLANTAGINIS HERBA	Cheqiancao	1.26 ± 0.62	5.46
14	FRITILLARIAE THUNBERGII BULBUS	Zhebeimu	1.82 ± 0.39	6.10
15	TRICHOSANTHIS RADIX	Tianhuafeng	0.91 ± 0.36	6.55
16	RUBIAE RADIX ET RHIZOMA	Qiancao	1.81 ± 0.66	5.83
17	CYNANCHI ATRATI RADIX ET RHIZOMA	Baiwei	1.67 ± 0.62	5.91
18	LEONURI HERBA	Yimucao	2.54 ± 0.84	6.09
19	CORYDALIS RHIZOMA	Yanhusuo	2.8 ± 0.4	6.59
20	STEPHANIAE TETRANDRAE RADIX	Fangji	3.01 ± 0.42	6.76
21	SCUTELLARIAE RADIX	Huangqin	3.28 ± 0.53	5.36
22	MENISPERMI RHIZOMA	Beidougen	2.03 ± 0.89	6.33
23	BLETILLAE RHIZOMA	Baiji	1.78 ± 0.92	4.57
24	SWERTIAE HERBA	Dangyao	3.92 ± 0.53	5.75
25	MELIAE CORTEX	Kulianpi	1.47 ± 0.84	6.78
26	NELUMBINIS PLUMULA	Lianzixin	3.47 ± 1.17	6.86
27	FRAXINI CORTEX	Qinpi	1.4 ± 0.63	6.18
28	COPTIDIS RHIZOMA	Huanglian	4.45 ± 0.77	7.70
29	SOPHORAE FLAVESCENTIS RADIX	Kushen	4.78 ± 0.63	6.91
30	GENTIANAE RADIX ET RHIZOMA	Longdan	4.55 ± 0.68	5.79
31	PHELLODENDRI CHINENSIS CORTEX	Huangbo	4.66 ± 0.69	6.56
32	BRUCEAE FRUCTUS	Yadanzi	1.1 ± 0.5	8.56
33	ANDROGRAPHIS HERBA	Chuanxinlian	4.04 ± 0.52	6.70
34	PICRORHIZAE RHIZOMA	Huhuanglian	4.67 ± 0.54	4.65
35	PICRASMAE RAMULUS ET FOLIUM	Kumu	4.08 ± 0.75	7.89

We prepared 10-fold concentrations of the 35 TCM samples relative to the prescribed dosage (e.g., the recommended dosage of *Coptidis Rhizoma* is 2–5 g, so the average value was 3.5 g). The TCM pieces were placed in 2000 mL water in an appropriate container, soaked for 30 min, and heated in a microwave (2100 W) until boiling. The power was then reduced to 600 W, and the solution was heated for another 20 min. The remaining herb pieces were filtered for the second decoction, after which an additional 2000 mL of water was added, the solution was heated until boiling, and then boiled for 10 min. The filtrates were combined, mixed, cooled to room temperature, and then centrifuged for 15 min at 4000 rpm. The supernatant was collected, and the volume adjusted to 4000 mL. The sample bottles were filled with this product, capped, sterilized, and stored at 4 °C. These were measured within 1 month.

### 2.4. Experiments

#### 2.4.1. THTPM and Data Processing

The methods of “gustatory sensation evaluation” and “outlier handling” in the present study are essentially the same as those described in a previous paper [27].

#### 2.4.2. E-Tongue Measurements

The samples were filtered, and then 80 mL was transferred into a 120 mL beaker for e-tongue testing, which was placed in the e-tongue autosampler tray for the measurement sequence. The signals initially fluctuated and then stabilized after 2–3 replicates. All of the samples were analyzed seven times, and each analysis cycle lasted 120 s. A value was collected every second, and the value generated at 120 s was used as the final output value. The values collected from the last four seconds were used as the raw data for principal component analysis. After measurement, the e-tongue was placed in a cleaning beaker. Before data collection, the e-tongue system was validated by self-testing, diagnosis, and calibration to confirm that the data were reliable and stable. To prevent errors caused by the recorded test sequence, the orders of different test samples were randomized.

### 2.5. Bitterness Evaluation

The 35 samples were tested using the seven sensors. The mean of the last four replicates was used as the response number corresponding to each sensor for a given drug. Finally, a 35 × 7 matrix was generated.

#### 2.5.1. RPLS Modeling

##### Optimization of Model Parameters

There were some outliers due to the subjective judgment of the THTPM. Thus, the robust component selection (RCS) statistic was used to select the optimal number of latent variables in the RPLS model:

(1) $$RCS_{k}=\sqrt{\gamma R-RMSECV_{k}^{2}+\left(1-\gamma \right)R-RMSE_{k}^{2}}$$

(2) $$RMSECV_{k}=\sqrt{\frac{1}{n}{\sum_{i=1}^{n}\left\|\left(y_{i}-{\hat{y}}_{-i,k}\right)\right\|}^{2}}$$

where, *k* is the number of latent variables, γ∈[0,1] is the parameter tuning the contribution of the quality of predictions (R-RMSE) in RSC, and ${\hat{y}}_{-i,k}$ is the cross-validated prediction of $y_{i}$ based on *k* latent variables. In contrast, ${\hat{y}}_{i,k}$ was obtained using all of the observations, including the *i*th sample. The calculation of R-RMSECV (robust RMSECV) was similar to that of RMSECV, but excluded outliers. By replacing ${\hat{y}}_{-i,k}$ with ${\hat{y}}_{i,k}$ the R-RMSE (robust RMSE) term was obtained. If *γ* was small, the goodness-of-fit (R-RMSECV) became the dominant one in the RCS statistic. Conversely, if *γ* was large, the quality of predictions contributed more to the RCS. When *γ* was 0.5, both the goodness-of-fit and quality of the predictions were given the same weight.

##### Modeling

After the number of latent variables was determined by the cross-validated procedure, a RPLS model was constructed using the e-tongue data to model the relationship between the electronic sensors and THTPM. This gave a robust RMSEP estimation.

##### Outlier Detection

The THTPM was particularly susceptible to outliers due to its subjective nature. Sample quality was examined based on parameters of the RPLS model. In this study, the examination of sample quality was realized by both the standardized residual (SR) and score distance (SD):

(3) $$Sr_{i}=r_{i}/s$$

(4) $$\text{where}\;r_{i}=y_{i}-{\hat{y}}_{i},\;s=\sqrt{\sum_{i=1}^{n}r_{i}^{2}/\left(n-p-1\right)}$$

(5) $$\text{and}\;SD_{i}=\sqrt{{\left(t_{i}-{\hat{\mu }}_{t}\right)}^{T}{\hat{\sum }}_{t}^{-1}\left(t_{i}-{\hat{\mu }}_{t}\right)}$$

${\hat{\mu }}_{t}$ and ${\hat{\sum }}_{t}$ were obtained from the RPLS model, the SD threshold was determined by c = $\sqrt{\chi_{k,0.975}^{2}}$, and the threshold of Sr was usually set to 2.5 σ.

The abovementioned distances defined three types of outliers: a good leverage point, a bad leverage point, and vertical outliers. The good leverage point referred to samples belonging to SD outliers with a small evaluation point, which were consistent with the linear trend of the overall sample. A bad leverage point referred to samples belonging to both the SD and Sr outliers. Vertical outliers only referred to the Sr outliers. After the sophisticated outliers were detected from the training set, the RPLS model was retrained. The training parameters remained the same, but with the fraction of outliers set to 0.

#### 2.5.2. Comparison to Other Models

Three other benchmark methods including MLR, LSSVM, and the plain PLS were used to correlate the bitterness determined by THTPM with data from the e-tongue. The cross-validation error was used as the index for optimizing the parameters of each method. Both R_CV_^2^ and RMSECV of all the models were recorded. Furthermore, all three methods were used to build a model using the dataset after the outliers were removed.

## 3. Results and Discussion

### 3.1. Parameter Optimization for RPLS

**Figure 1:**
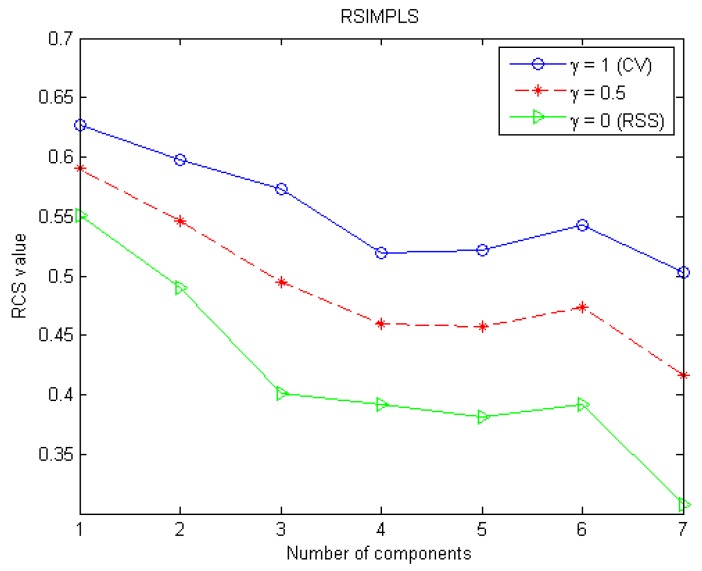
Optimization the number of latent variables (components).

The optimization result of the number of latent variables is showed in Figure 1. The “CV” in parentheses after lambda = 1 means that only the contribution of the quality of predictions remains in the RCS. Similarly, the “RSS” in the parentheses after lambda = 0 indicates that only the good-of-fit remains in the RCS. With three different setting on the tuning parameter lambda (γ = 1, 0.5, 0), the RCS as a function of increasing number of latent variables showed a similar behavior with a plateau after four variables. Therefore, only four latent variables remained in the final predictor.

### 3.2. Outlier Detection

**Figure 2:**
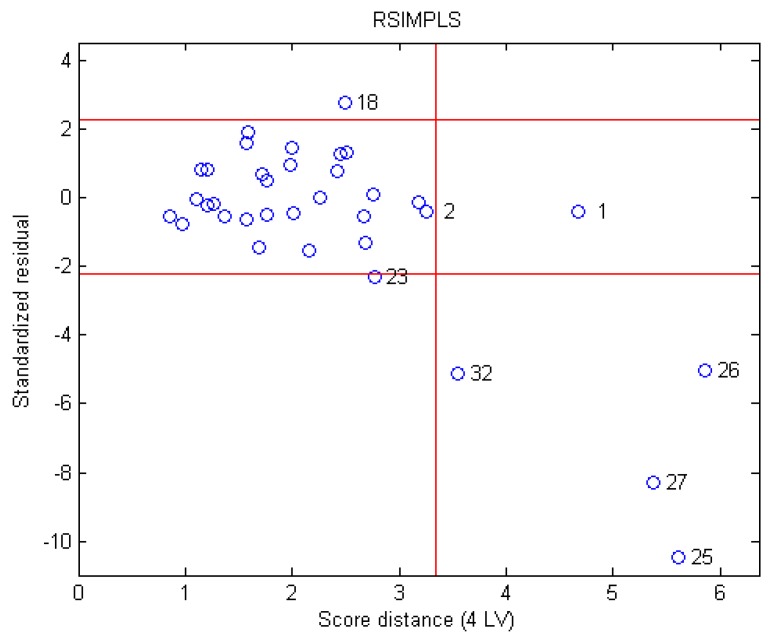
Distribution of all the samples in the space spanned by Standardized residual and Score distance.

Based on the parameters of the RPLS model, the Sr and SD of each sample were calculated. The Sr was plotted against SD (Figure 2). Sample 1 had a good leverage point. However, it had a positive influence on the bitterness quantitative model, thus, it was not removed from the dataset. Samples 18, 25, 26, 27, and 32 were excluded when building the PLS model. To ensure a high-quality evaluation, sample 23 (located on the border) was also discarded.

After carefully examining the six outliers (*i.e.*, samples 18, 23, 25, 26, 27, and 32), it was found that the outliers had the following features in common that resulted in their exclusion: (1) The outliers had sensor values that greatly exceeded those of all other samples. This caused poor extrapolation of the model; (2) Samples with strong bitterness may leave residual bitterness in the next test. This might be due to insufficient cleaning or the test order; and (3) Certain samples had markedly different pH values.

### 3.3. Bitterness Predictor Constructed Using RPLS

**Figure 3:**
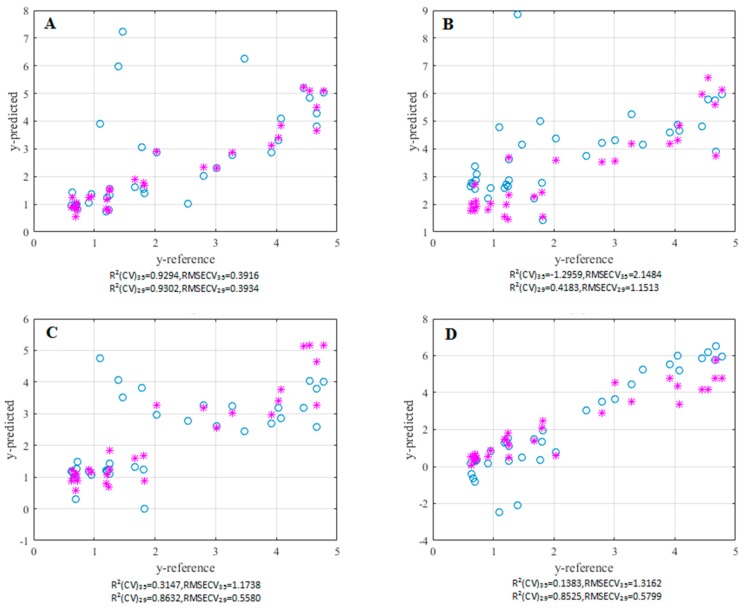
Comparison of the prediction performances of all four methods (**A**) RPLS; (**B**) MLR; (**C**) PLS; (**D**) LS-SVM. Here, ○ represents before excluding outliers, * represents after excluding outliers.

The model established using RPLS based on the entire dataset was transformed into its original variable form:

(6) *Ii* = (−3.2322e−4) × ZZ + (4.1632e−4) × BA – (4.7319e–4) × BB + 0.00253 × CA + 0.00225 × GA + (1.8467e–4) × DA + (4.3317e–5) × AB − 4.3613

where, *Ii* is the estimated bitterness intensity, ZZ, BA, BB, CA, GA, DA, and AB are the original variables of the seven sensors, and −4.3613 is the intercept. The estimated results are shown in Figure 3 with an R^2^ of 0.9002 and a robust RMSEP of 0.5421. This indicates that the model had good evaluation performance. For the mode built using RPLS on the 29 remaining samples, an R_CV_^2^ of 0.9302 and a RMSECV of 0.3934 were obtained. The RPLS model constructed on the whole dataset showed an R_CV_^2^ of 0.9394 and a RMSECV of 0.3916. Therefore, without an extra requirement on outlier detection, the prediction performance of RPLS on the remained dataset will be the same as those on the raw dataset.

### 3.4. Comparisons with Other Modeling Methods and Between Pre-Screening and Post-Screening Models

#### 3.4.1. MLR

MLR models were built for all 35 samples and the 29 samples selected by RPLS. The resulting R_CV_^2^ and RMSECV values calculated by leave one out cross validation (LOOCV) are shown in Figure 3. The outliers seriously damage the prediction performance of MLR. After six stray samples were removed from the training set, the prediction performance was improved markedly, but its performance was still far worse than that of the RPLS.

#### 3.4.2. PLS

The PLS method is specialized in dealing with collinear, and was used to construct a predictor for bitterness. From the R_CV_^2^ and RMSECV recorded in Figure 3, it can be observed that the improvement in the model built on the whole dataset was obvious compared to the MLR. After the outliers were removed, the PLS model presents a R_CV_^2^ value of 0.8632, which is close to but still worse than that of RPLS. Thus, it can be concluded that alterations in the approach to estimate the covariance matrix and the variance matrix is not only helpful in detecting outliers, but also improves the prediction performance of PLS.

#### 3.4.3. LSSVM

Based on the R_CV_^2^ and RMSECV values estimated by LOOCV, the RBF kernel LSSVM was selected to predict the bitterness of herbs. The results in Figure 3 clearly showed that although LSSVM is a sound non-linear learning method, it is still sensitive to outliers. When the outliers were removed, the prediction performance of LSSVM was comparable to that of PLS, but was still worse than RPLS.

Indeed, the prediction performance of the model constructed after the outliers removed were all better than those built using the raw dataset.

## 4. Conclusions

To evaluate the bitterness of Traditional Chinese Medicines, 35 TCM decoctions whose bitterness is clearly specified in Chinese Pharmacopoeia (version 2010) were used. The human-based THTPM served as the reference to evaluate and quantify the intensity of bitterness. The cross validation results showed RPLS could be used to quantitatively characterize bitterness, since the RPLS model presented a robust RMSECV value of 0.3916 with bitterness values ranging from 0.63 to 4.78 on the entire dataset. After comparing among MLR, LSSVM, and PLS regression models on pre-screening and post-screening samples, we found that outlier exclusion largely improved the evaluation performance. But the difference between the RPLS model constructed before and after outlier exclusion was negligible. Thus it can be concluded that by using the RPLS method along, a usable model can be constructed to quantify the bitterness of TCMs.
